# Intraoperative Myocardial Infarction During Elective Laparoscopic Cholecystectomy: A Case Report

**DOI:** 10.7759/cureus.76004

**Published:** 2024-12-19

**Authors:** Amr Barghout, Bethany Plummer

**Affiliations:** 1 Anaesthesia, Medway NHS Foundation Trust, Kent, GBR

**Keywords:** abdominal insufflation, co2 pneumoperitoneum, haemodynamic compromise, laparoscopic chole, myocardia infarction

## Abstract

Laparoscopic cholecystectomy has become the gold standard for treating symptomatic cholelithiasis due to its minimally invasive nature and faster recovery times compared to traditional open surgery, but it is not without risks. A key component of this procedure is the creation of pneumoperitoneum. This is achieved by insufflating the abdomen with carbon dioxide (CO2). This process causes an increased intra-abdominal pressure (IAP), reduced venous return, and disrupted myocardial oxygen supply and demand. These changes may predispose vulnerable patients to acute cardiac events, particularly those with underlying cardiovascular risk factors.

We present a case study of an intraoperative myocardial infarction (MI) that occurred during an elective laparoscopic cholecystectomy. During the procedure, the patient exhibited acute haemodynamic instability (bradycardia and hypotension). Electrocardiographic findings and cardiac biomarkers later confirmed the diagnosis. Immediate intraoperative interventions, including stabilisation of haemodynamics and removal of pneumoperitoneum. Postoperative recovery was monitored in the intensive care unit (ICU), with subsequent investigations identifying pre-existing but previously undiagnosed coronary artery disease as a contributing factor.

This case highlights the importance of thorough preoperative cardiovascular evaluation, particularly in patients with risk factors for coronary artery disease, even for procedures considered minimally invasive. It also stresses the potential role of pneumoperitoneum in precipitating acute cardiac events due to its significant haemodynamic impact. Timely intraoperative management, combined with prompt access to cardiology expertise, evaluation, and invasive interventions, is essential for optimising patient outcomes in such cases.

## Introduction

Laparoscopic cholecystectomy is a minimally invasive surgical procedure used for the removal of a diseased gallbladder and is recommended for most patients with symptomatic gallstones [1]. Laparoscopic cholecystectomies have gained widespread acceptance since the early 1990s, with over 75% being performed as a day case. Today, it is considered the preferred technique by almost all surgeons [2]. About 66,660 cholecystectomies are performed in the UK annually, of which the majority are laparoscopic, costing the NHS about £111 million [3]. Between April 2005 and April 2010, 11 deaths and 37 serious incidents were reported to the National Patient Safety Agency (NSPA) in patients who had deteriorated after laparoscopic surgery, and arguably this is likely to massively under-represent the true number [4].

During laparoscopic procedures, the creation of pneumoperitoneum by carbon dioxide (CO2) insufflation is the most widely accepted technique for adequate working space and patient safety [5].

The most common approach for laparoscopic cholecystectomy uses a four-port technique with two 10 mm diameter ports, one in the umbilical and another in the epigastrium areas, and two 5 mm diameter right upper quadrant ports. There are several variations in this technique. The standard operating pressure is up to 12 mmHg to maintain the CO2 pneumoperitoneum [6].

As the volume of the abdomen increases, abdominal wall compliance decreases, and intra-abdominal pressure (IAP) rises. Systemic vascular resistance (SVR) is increased due to both mechanical compression of the abdominal aorta and activation of the neurohormonal stress response, such as activation of the renin-angiotensin-aldosterone axis. These factors increase the afterload and myocardial oxygen consumption, which are poorly tolerated by patients with cardiac dysfunction [7].

Elevated IAP and reverse Trendelenburg position can cause compression of the inferior vena cava, reducing preload and may lead to a decrease in cardiac output, specifically if the patient is hypovolemic [8]. This combination of decreased preload and increased afterload increases cardiac workload and could precipitate cardiac ischaemia or infarction. Patients with ischaemic heart disease are prone to developing atrial fibrillation, a condition that could be precipitated by CO2 pneumoperitoneum [9].

## Case presentation

A 67-year-old male patient was booked for a day-case laparoscopic cholecystectomy due to symptomatic cholelithiasis and a prior episode of acute cholecystitis four months earlier.

He had a BMI of 28.7 kg/m², a background of hypertension that is well controlled by a single agent (amlodipine) as per National Institute of Clinical Excellence (NICE) guidance, and he was an ex-smoker (10 pack-year history). Family history of cardiomyopathy and type 2 diabetes mellitus. Three years prior, he underwent echocardiography, which revealed normal findings except for left ventricular hypertrophy, consistent with his known hypertension. He had no known drug allergies. Preoperative ECG showed a normal sinus rhythm at 56 beats per minute (bpm) and moderate voltage criteria suggestive of left ventricular hypertrophy, categorising him as an American Society of Anaesthesiologists (ASA) grade II patient.

On arrival at the anaesthetic room, the Association of Anaesthetists of Great Britain and Ireland (AAGBI) standards of monitoring were attached. The patient’s blood pressure was 140/85 mmHg with a heart rate of 66 bpm. A peripheral cannula (18 gauge) was inserted. Preoxygenation and a high-dose opioid, 200 mcg fentanyl, were used to mitigate laryngoscopy and intubation-induced stress response as we identified the patient had risk factors of cardiac events (hypertension, family history). General anaesthesia (GA) induction was unremarkable, with further administration of 150 mg + 50 mg propofol, 50 mg rocuronium, 4 mg ondansetron, and 6.6 mg dexamethasone. An endotracheal tube (size 8) was inserted without complications. The patient remained haemodynamically stable throughout induction and was transferred to the operating room. Following induction, the blood pressure was recorded at 122/79 mmHg and the heart rate at 60 bpm.

Shortly after the induction of pneumoperitoneum with carbon dioxide at a pressure of 12 mmHg, the patient experienced sudden hypotension (62/30 mmHg) and bradycardia (initially 35 bpm). A bolus of 600 mcg atropine and 9 mg ephedrine was administered with no response. The surgeon was instructed to immediately deflate the pneumoperitoneum. Despite this, the heart rate continued to drop, at its lowest 25 bpm, necessitating a bolus of 100 mcg adrenaline. The heart rate then surged to 120 bpm, and blood pressure spiked to 210/110 mmHg before gradually normalizing. Concurrently, the patient experienced acute desaturation from 98% to 88%. The fraction of inspired oxygen (FiO2) was increased to 100%, and bag-valve ventilation was initiated. Frothy secretions were noted in the endotracheal tube and suctioned, raising the suspicion of pulmonary oedema.

**Figure 1:**
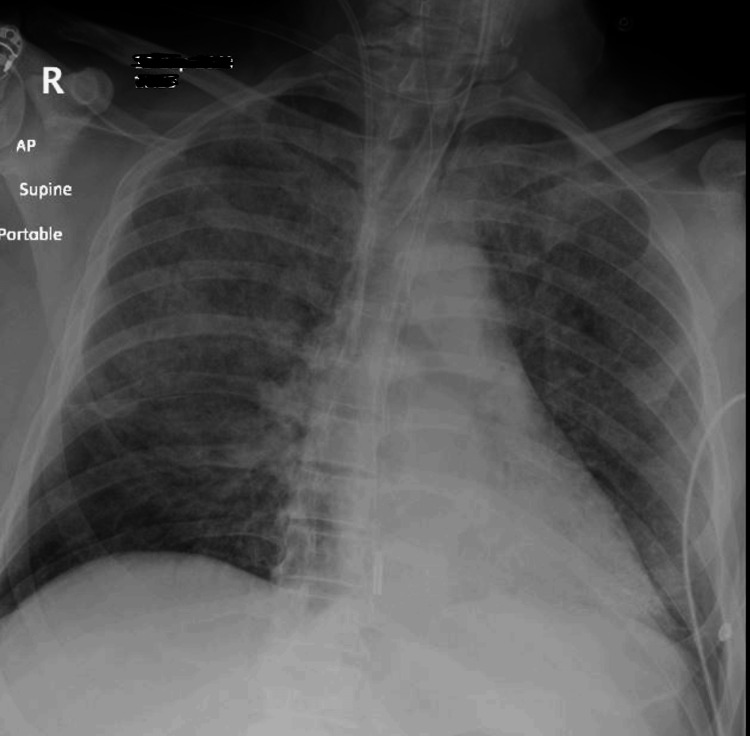
Portable chest X-ray in theatre showing bilateral lung opacifications

A nitroglycerin infusion was initiated, and both an arterial line and a central venous catheter were placed to facilitate invasive monitoring and provide vasopressor support via noradrenaline infusion. The ECG monitor showed ST depression in leads II and III, findings that were confirmed on a newly obtained 12-lead ECG, which additionally revealed ST depression in leads V1 and V2. Arterial blood gas analysis indicated severe type 1 respiratory failure, with a partial pressure of oxygen (PaO2) of 7.2 kPa on 100% oxygen, a partial pressure of carbon dioxide (PaCO2) of 5.8 kPa, and an elevated lactate level of 2 mmol/L. A chest X-ray revealed bilateral lung opacifications (Figure 1).

A bedside echocardiogram showed marked dilation of the right side of the heart with preserved function and fair contractility of the left ventricle. After initial stabilisation, the patient was transferred for a CT pulmonary angiography to exclude pulmonary embolism, followed by transfer to the intensive care unit (ICU) for further management.

**Figure 2:**
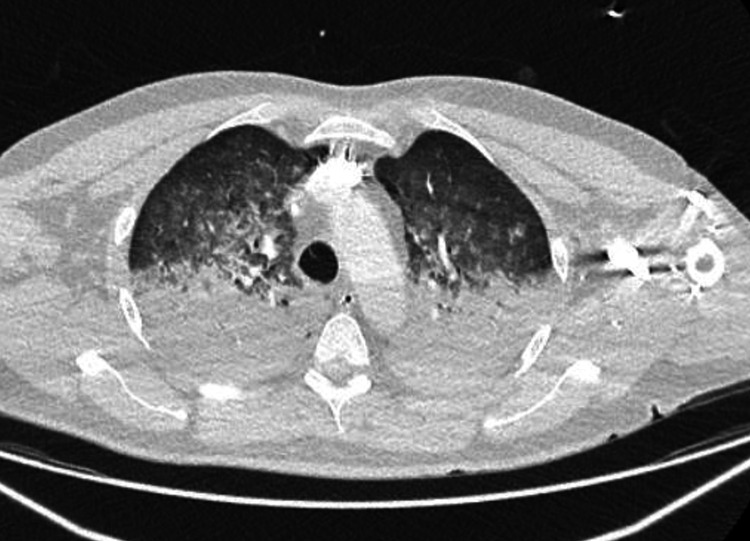
A CT scan of the chest before ICU admission showing bilateral lung extensive consolidations

The CT pulmonary angiography scan was negative for blood clot pulmonary embolism but revealed extensive bilateral consolidations in both lower lobes (Figure 2) suggestive of pulmonary oedema as more basal and bilateral. In the ICU, the patient's troponin levels trended upwards from 3000 to 7000 ng/L, prompting the initiation of the local acute coronary syndrome treatment protocol. The patient clinically improved was extubated two days later and transferred to the coronary care unit (CCU) a week later. In the CCU, he underwent inpatient coronary angiography, which revealed severe multivessel disease. There was evidence of moderate to severe osteal disease in the left main stem, severe proximal disease in the left anterior descending artery, severe disease in diagonal branches, and a completely occluded posterior left ventricular artery in the right coronary artery. He was advised to undergo coronary artery bypass grafting (CABG). The patient was discharged home and scheduled for outpatient CABG at a tertiary centre.

## Discussion

According to the American College of Surgeons National Surgical Quality Improvement Program (ACS NSQIP), this patient had a predicted adverse cardiac event risk of 0.1%. Similarly, the Revised Cardiac Risk Index (RCRI) calculated a 3.9% risk of major cardiac events, classifying him as low risk. The patient had a good functional status and no symptoms suggestive of ischaemic heart disease, aligning with a good preoperative profile.

Despite American Heart Association (AHA) guidelines not recommending a routine preoperative ECG, local practice is for all individuals over 65 to have an ECG; an ECG was performed and revealed left ventricular hypertrophy. According to AHA, this finding could have warranted further cardiac evaluation, such as echocardiography and cardiac biomarkers. These additional investigations may have provided deeper insights into underlying cardiovascular conditions, although on balance unlikely.

Perioperative myocardial infarction is one of the most important predictors of short- and long-term morbidity and mortality associated with non-cardiac surgery [10]. At one year, mortality for perioperative MI and for myocardial injury (troponin elevation alone) was 22% in a recent study [11].

The pathophysiology of intraoperative MI in this context may be down to several factors: underlying coronary artery disease, anaesthesia-induced myocardial depression, anaesthesia-induced hypotension, the sudden increased IAP leading to myocardial oxygen supply-demand mismatch, and gas embolism with right ventricular failure.

In this case, the patient exhibited sudden hypotension and bradycardia following insufflation of the abdomen. Intraoperative ECG changes, including ST depression, provided further evidence of myocardial ischemia. Initial management included haemodynamic stabilisation; atropine and ephedrine were administered to address bradycardia and hypotension. When deterioration continued, adrenaline was used to avert imminent cardiac arrest with limited benefit. Removing the pneumoperitoneum relieved the increased IAP, improving venous return and cardiac output. Acute desaturation with frothy endotracheal secretions indicated pulmonary oedema, managed with increased FiO2 and suctioning. This was likely due to the acute onset of cardiogenic pulmonary oedema. The sudden rise in left-sided intra-cardiac filling pressures in the setting of acute ischemia. It is therefore likely that the intraoperative IAP caused the SVR to increase, increasing the afterload and myocardial oxygen consumption. With the multiple diseased vessels, we were not able to achieve the required coronary perfusion pressure, and cardiogenic shock occurred.

Postoperative findings of elevated troponin levels and ECG abnormalities confirmed myocardial injury. Coronary reperfusion planning is fundamental in preventing further coronary events for this patient. 

## Conclusions

This case discusses the balance between adhering to risk-based screening guidelines and the potential benefits of individualised decision-making in patients undergoing laparoscopic procedures with predisposing cardiovascular risk factors. Adapting perioperative guidelines to account for the haemodynamic stresses recognised in laparoscopic surgeries may improve patient safety. Areas for further research include developing predictive tools for those with undiagnosed coronary artery disease and those having laparoscopic surgeries and investigating alternative methods to mitigate pneumoperitoneum-induced haemodynamic changes without compromising surgical outcomes. For example, introducing the “sip until send” campaign to reduce the risk of hypovolaemia.

This case contributes valuable insights into the interplay between pneumoperitoneum-induced haemodynamic changes and cardiac ischaemia. It reinforces the necessity for nuanced clinical decision-making and provides an argument for refining perioperative cardiac investigations policies in laparoscopic surgeries.
